# Malaria and Anemia among Children in a Low Resource Setting In Nigeria

**Published:** 2012

**Authors:** BH Oladeinde, R Omoregie, M Olley, JA Anunibe, AA Onifade, OB Oladeinde

**Affiliations:** 1 Department of Medical Microbiology, College of Health Sciences, Igbinedion University, Okada, Edo State, Nigeria; 2 School of Medical Laboratory Sciences, University of Benin Teaching Hospital, Edo State, Nigeria; 3 Department of Pathology, Igbinedion University Teaching Hospital, Okada, Edo State, Nigeria; 4 Department of Chemical Pathology, College of Health Sciences, Igbinedion University, Okada, Edo State, Nigeria; 5 Faculty of Health & Social Care, St George's University of London & Kingston University London; 6 Department of Obstetrics and Gyneacology, Irrua Specialist Hospital, Irrua Edo State. Nigeria

**Keywords:** Malaria, Anemia, Insecticide treated bed nets, Rural community, Nigeria

## Abstract

**Background:**

This study aimed at determining the prevalence of malaria and anemia among children in rural community of Okada, Edo State Nigeria, as well as to assess the level of use of Insecticide treated bed nets and its impact on prevalence of malaria and anemia among study population.

**Methods:**

Thick blood films from 226 children with signs and symptoms of malaria in Okada community were stained and examined for presence of malaria parasites. Hemoglobin concentration of all children was also determined using standard method.

**Result:**

A total of 185 (81.9%) children were infected with malaria parasite. Malaria parasitaemia was significantly affected by age (*P* =0.003). A significantly higher number of positive cases of malaria and anemia was observed in rainy season as compared to dry season (*P*<0.05). The prevalence of anemia in children was 47.3%. Malaria was a risk factor for development of anemia in children (OR=2.551; 95% CI=1.227, 5.305; *P*=0.015). Use of insecticide treated bed nets was recorded in 11(4.9%) of children studied, and did not significantly reduce the prevalence of malaria and anemia. However among malaria parasite infected children, its use significantly reduced the prevalence of anemia (OR=0.126; 95%CI=0.015, 1.047; *P*=0.031).

**Conclusion:**

Malaria and anemia among children was high malaria intervention progammes by relevant agencies is strongly advocated.

## Introduction

Malaria is a leading cause of morbidity and mortality worldwide. Data from World Health Organization shows that approximately 216 million cases of clinical malaria occur worldwide, with deaths of about 655 000 occurring most among African children (1). Every 45 seconds, one child in Africa dies of malaria (2). Recent world malaria report indicates that Nigeria accounts for a quarter of all malaria cases in the 45 malaria endemic countries in Africa (3). Malaria transmission is higher in rural areas than urban settlements in Africa (4, 5). Factors such as lower vector density, higher human density, better and quality housing, improved drainage systems and relative ease of assessing healthcare facilities in urban areas are reasons for the observed lower prevalence of malaria in urban settings (4, 6). The aforementioned variables are virtually non existent in most rural communities of Nigeria. The use of Insecticide Treated Bed Nets (ITN), have been found to have a protective efficacy against malaria episodes of approximately 50% in highly endemic areas of Africa (7) and have also been shown to reduce overall mortality in children by 63% in villages using impregnated nets (8).

Anemia is a known complication of malaria. It has a profound effect on the quality of life of people by inducing such symptoms as loss of stamina, rapid heart rate and shortness of breath (9). It has also been reported that over half of malaria related deaths are attributable to severe anemia (10). Ignorance, poverty and gender bias, also significantly contribute to high prevalence of anemia (11). These factors are rife in Okada community of Edo State and indeed most other rural settings in Nigeria. Although few studies exist on malaria and anemia among children in Nigeria, none have focused on strictly rural populations and assessed the level of use and impact of ITN on the prevalence of malaria and anemia. Against this background this study focused on determining the prevalence of malaria and anemia among children in rural Okada community of Edo state, as well as to ascertain level of use of ITN and its impact on prevalence of malaria and anemia.

## Materials and Methods

### Study area

Okada, a rural community, is the headquarters of Ovia North East Local Government Area of Edo-state. The Local Government has an estimated population of 155 344 persons (12). Majority of the residents of Okada are Farmers with few Civil Servants, Lecturers and students making less than 5% of the community. The study was carried out at Igbinedion University Teaching Hospital. Okada, Edo State, Nigeria, from April 2010 to March 2011. Some neighboring rural communities (villages) also attend the Hospital.

### Study population

A total of 226 children (132 males and 94 females), at the out-patient department of the Igbinedion University Teaching Hospital, presenting with signs and symptom of malaria were recruited for this study. The age range of study population was 0.17 – 10 years. Verbal informed consent was obtained from parents/guardians of all participating children prior to specimen collection. Data on age and use of Insecticide treated bed nets was obtained from parents /guardian of all participating children. The study was approved by the Ethical Committee of the Igbinedion University Teaching Hospital, Okada, Nigeria.

### Collection and processing of specimen

About five ml of blood was collected from each subject and dispensed into ethylene diamine tetra-acetic acid (EDTA) container and mixed. Malaria was diagnosed by examination of stained thick blood films. Thick blood films were made from each blood sample and allowed to air dry. Slides were stained in 3% Giemsa stain for 30 minutes, rinsed in tap water, and allowed to air dry. The stained films were examined for malaria parasite by microscopy using a × 100 oil immersion objective lens. A total of 200 fields per film were examined. Hemoglobin concentration was determined using an autoanalyser – Sysmex KX-21 (Sysmex Corporation, Kobe, Japan). Anemia was defined a hemoglobin concentration <11g/dl (13).

### Statistical analysis

The data obtained were analyzed using Chi-square (X^2^) or Fischer's exact test as appropriate and odd ratio analysis using the statistical software INSTAT^®^. Statistical significance was set at *P* < 0.05

## Results

A total of 185(81.9%) out of the 226 examined were infected with malaria parasite. Age was a risk factor for infection with malaria parasite, with children within the age group of =1-2 years having the highest prevalence of 90.0%, (*P*=0.003). A significantly higher number of malaria parasite positive cases was observed during the rainy season (OR=2.486; 95%CI=1.235, 5.006; *P*=0.0152). Gender and use of Insecticide treated bed nets (ITN) did not significantly affect the prevalence of malaria parasite infection in children. Use of Insecticide treated bed nets was recorded in 11(4.9%) of children (Table 1).

**Table 1:** Prevalence of malaria among children

Characteristics	No.	No. Positive (%)	OR	95%CI	*P* value
**Season**					
**Rainy**	124	109(87.9)	2.486	1.235, 5.006	0.015
**Dry**	102	76(74.5)	0.402		
Gender					
**Male**	132	114(86.4)	2.052	1.035, 4.068	0.056
**Female**	94	71(75.5)	0.487		
Age (Years)					
**≤ 1-2**	40	36 (90.0)			0.003
3-4	66	57 (86.3)			
**5-6**	35	29 (82.8)			
**7-8**	51	42 (82.4)			
**9-10**	34	21 (61.8)			
Use of ITN					
**Yes**	11	8 (72.7)	0.573	0.145, 2.259	0.424
**No**	215	177(82.3)	1.747		

N=number tested; OR=odd ratio; CI=confidence interval; ITN –insecticide treated bed net

The prevalence of anemia in children among children was 47.3%. A significantly higher prevalence of anemia was recorded among children in the rainy season (OR=1.962; 95% CI=1.150, 3.344; *P*=0.019). Gender, age, and use of Insecticide treated bed nets (ITN) did not significantly affect the prevalence of anemia in children (Table 2).

**Table 2:** Prevalence of anemia among children

Characteristics	No.	No. Anemic (%)	OR	95%CI	*P* value
**Season**					
**Rainy**	124	68(54.8)	1.962	1.150, 3.344	0.019
**Dry**	102	39(38.2)	0.509		
Gender					
**Male**	132	56(42.4)	0.621	0.365, 1.053	0.105
**Female**	94	51(54.3)	1.610		
Age (Years)					
**=1-2**	40	16(40.0			0.163
3-4	66	27(40.9)			
**5-6**	35	18(51.4)			
**7-8**	51	23(45.1)			
**9-10**	34	23(67.6)			
Use of ITN					
**Yes**	11	4(36.4)	0.621	0.176, 2.185	0.5452
**No**	215	103(47.9)	1.609		

N=number tested; OR=odd ratio; CI=confidence interval; ITN –insecticide treated bed net

The prevalence of anemia in malaria parasite infected children was 51.4%. Malaria was a risk factor for development of anemia in children (OR=2.551; 95% CI=1.227, 5.305; *P*=0.015).Among malaria parasite infected children, the use of Insecticide treated bed nets (ITN) was found to significantly reduced the prevalence of anemia (OR=0.126;95%CI=0.015, 1.047; *P*=0.031) (Table 3).

**Table 3:** Effect of malaria parasitaemia and ITN use on prevalence of anemia

Characteristics	No.	No. Anemic (%)	OR	95%CI	*P* value
Malaria status					
**Positive**	185	95(51.4)	2.551	1.227, 5.305	0.015
**Negative**	41	12(29.3)	0.392		
Malaria status					
**Positive (with ITN use)**	8	1(12.5)	0.126	0.015, 1.047	0.031
**Positive (without ITN use)**	177	94(53.1)	7.928		

N=number tested; OR=odd ratio; CI=confidence interval; ITN=insecticide treated bed nets.

## Discussion

Despite recent advances made in malaria prevention and control globally, malaria still remains a major health concern in Sub Saharan Africa with very high mortality rates being recorded among children. This study focused on determining the prevalence of malaria and anemia among children in rural community of Okada, Edo State Nigeria, as well as the level of use of Insecticide treated bed nets and its impact on prevalence of malaria and anemia.

Malaria parasite infection was recorded among 185(81.9%) of children studied. This is higher than findings from other African studies (13–17). Differences in environmental factors, existing malaria intervention and prevention strategies, and class of children examined, may be responsible for the observed variation. *Plasmodium falciparum* was the only specie of malaria parasite found in this study. The finding that malaria prevalence was significantly higher in rainy season in this study has been previously documented (18). Collections of water after heavy rainfall during the rainy season can serve as favorable breeding places for vectors of malaria. Bushes which are common sites around dwelling places in Okada are often left to grow out of proportion in rainy seasons creating a niche for larval proliferation in the community.

Age was a significant risk factor for malaria parasite infection among children. This is consistent with an earlier report (19). Children <1-2 years were mostly at risk of acquiring malaria. Elsewhere severe malaria tends to occur in older children (20). Differences in the age of presentation of severe malaria may be the result of lower background immunity or other unidentified variables (21).

The prevalence of anemia among children was 47.3%. This is lower than a finding in Benin City (13), and higher than figures reported in other African studies (14–16). An important factor to consider is that the etiology of anemia is multifactorial, and thus several underlying morbid and co- morbid conditions could cause wide variations in prevalence of anemia among children in different clinical settings. Anemia was significantly higher in the rainy season than dry season. The endemicity of malaria in this area during the rainy season coupled with the general worsening economy in Nigeria may account for this observation. Use of insecticide treated bed net by children in rural Okada community was poor (4.9%). This is lower than findings in other Nigerian studies (22, 23). Widespread distribution of free insecticide treated bed net in Nigeria is still a dream unlike other African countries where wide coverage among the people has been reported (24, 25). The high cost of its purchase amongst other factors may account for its low use in this study. Although proven to be effective in reduction of malaria and anemia (26, 27), the use of ITN did not significantly reduce the prevalence of malaria and anemia among children studied. This is consistent with a previous finding (16). Low, infrequent, and improper use of ITN in a malaria endemic community like Okada, may have contributed to its seeming non-efficacy in study population.

Malaria was strongly associated with anemia in this study. This has been earlier reported (16). Interestingly however, among malaria parasite infected children, the use of ITN was found to significantly reduce the prevalence of anemia. The improvement in hemoglobin level may be associated with significant reduction of mosquito contact with children using the malaria prevention tool. Again, Insecticide treated bed nets are expensive to procure and not freely distributed in Ovia North East Local Government Area of Edo State. Some studies have found that higher socio economic status is related to ownership and use of ITN (28). It is possible that children in this study in possession and use of ITN may very well have been from well to do homes, with better nutritional status than those who did not use the malaria intervention tool. The history of previous malaria attacks which could have profound effect on anemia and malaria could not be measured. Sample size for this study was small, owing to low compliance rate. Again the effect of asymptomatic malaria cases on prevalence of anemia was not ascertained. These were limitations observed to the study.

Conclusively, the prevalence of malaria and anemia among children in Okada was 81.9% and 47.3% respectively. Use of insecticide treated bed nets was poor and did not significantly reduce malaria and anemia generally among children studied. Its use however was found to significantly reduced anemia among malaria infected children. Community based malaria intervention progammes, targeted at enlightenment on modes of malaria transmission, provision of ITNs, prompt identification and treatment of all cases are needed to stem malaria and associated squeal.
